# Incidence of Internal and Petersen's Hernias Following Gastrectomy for Gastric Cancer: A Meta‐Analysis of Surgical Approach and Preventive Closure

**DOI:** 10.1002/wjs.70257

**Published:** 2026-02-10

**Authors:** Sang‐Ho Jeong, Rock Bum Kim, Miyeong Park, Kyung Won Seo, Jae‐Seok Min

**Affiliations:** ^1^ Department of Surgery Gyeongsang National University College of Medicine and Gyeongsang National University Changwon Hospital Changwon Republic of Korea; ^2^ Regional Cardiocerebrovascular Disease Center Gyeongsang National University Hospital Jinju Republic of Korea; ^3^ Department of Anesthesiology Gyeongsang National University College of Medicine and Gyeongsang National University Changwon Hospital Changwon Republic of Korea; ^4^ Department of Surgery Kosin University Gospel Hospital Busan Republic of Korea; ^5^ Division of Foregut Surgery Korea University Anam Hospital Seoul Republic of Korea; ^6^ Department of Surgery Korea University College of Medicine Seoul Republic of Korea

**Keywords:** complication, hernia, laparoscopy, stomach neoplasms, surgery

## Abstract

**Background:**

Gastric cancer continues to pose a significant global health burden, with gastrectomy being the primary curative treatment. However, the increased performing of laparoscopic gastrectomy (LG) has been associated with a rising incidence of postoperative internal hernia (IH), particularly Petersen's hernia (pH), which may lead to bowel strangulation and necrosis. This meta‐analysis aimed to compare the incidence of IH following LG versus open gastrectomy (OG) and to evaluate the preventive effect of Petersen's space closure on pH occurrence.

**Methods:**

A systematic review and meta‐analysis were conducted using PubMed and Embase to identify studies published in the past 25 years that reported IH or pH after gastrectomy for gastric cancer. Eligible studies compared (1) the incidence of IH between LG and OG or (2) the incidence of pH between closure and nonclosure of Petersen's space. Pooled odds ratios (ORs) with 95% confidence intervals (CIs) were calculated using a random‐effects model.

**Results:**

Five studies comparing LG and OG demonstrated a significantly higher risk of IH in the LG group (OR 2.81, 95% CI: 1.40–5.62). A subgroup analysis limited to total gastrectomy showed a nonsignificant trend toward increased IH risk after LG (OR 6.12). Additionally, five studies showed that closure of Petersen's space significantly reduced the risk of pH (OR 5.73, 95% CI: 1.59–20.67).

**Conclusion:**

Laparoscopic gastrectomy is associated with an increased risk of internal hernia compared to open surgery for gastric cancer. The preventive closure of Petersen's space should be considered mandatory, particularly during Roux‐en‐Y reconstruction after gastrectomy.

## 1 Introduction

While internal hernia (IH) is a relatively rare postoperative complication after gastrectomy, it is often associated with potentially deleterious consequences, including bowel strangulation and ischemia. Furthermore, the true incidence of IH is likely underestimated, as diagnoses are predominantly made in symptomatic patients, leaving asymptomatic cases undetected [1]. Radical gastrectomy remains the cornerstone of treatment for patients with gastric cancer, as minimally invasive gastrectomy approaches (laparoscopy or robot) may lead to fewer postoperative adhesions, which, in turn, can result in a higher incidence of internal hernia than open surgery [2]. IH occurs in approximately 1.7%–4.9% of patients following gastrectomy [2]. Among these, Petersen's hernias (PHs), reported in 3.3% of patients following Roux‐en‐Y reconstruction, can rapidly lead to bowel strangulation, with intestinal necrosis developing in 36.4% of cases, which is correlated with a 27.3% mortality rate [3]. Although abdominal computed tomography (CT) detects whirlpool signs suggestive of IH in 72.7% of cases, nonspecific symptoms (epigastric pain 90.9%, vomiting 63.6%) often delay diagnosis [4].

Prophylactic closure of Petersen's space reduces hernia incidence to 0.4%, yet technical heterogeneity in defect closure (absorbable vs. nonabsorbable sutures) and a 6.93‐fold higher risk in laparoscopic versus open surgery underscore the urgent need for standardized protocols [4]. The long‐term outcome data are still insufficient, and preventive strategy optimization is critical as minimally invasive gastrectomy becomes predominant [2].

This meta‐analysis aimed to compare the incidence of IH between laparoscopic gastrectomy (LG) and open gastrectomy (OG) and to evaluate the effect of Petersen's space closure on pH occurrence.

## 2 Methods

### 2.1 Search Scheme and Selection of Studies

This systematic review and meta‐analysis were conducted via the PubMed, Embase and Cochrane Library databases to identify relevant studies published between January 2000 and December 2024 that investigated internal hernias following gastrectomy in patients with gastric cancer. The initial search yielded 112 articles via the keywords ((“stomach neoplasms”[MeSH] OR “stomach cancer” OR “gastric cancer”) AND (“gastrectomy”[MeSH] OR “gastrectomy” OR “gastric resection”)) AND (“laparoscopy”[MeSH] OR “laparoscopy” OR “laparoscopic” OR “open surgery” OR “open gastrectomy” OR “Petersen”) AND (“internal hernia”[MeSH] OR “internal hernia” OR “internal herniation” OR “hernia”)) “stomach surgery”, “internal hernia”, and “gastric cancer”.

After 81 articles obtained in the search that were not clinical trials or case report were removed, a total of 31 studies remained. Two authors (S.‐H. Jeong and J.‐S. Min) independently screened the titles and abstracts of these 31 studies, coding them as ‘retrieve’ (eligible, potentially eligible, or unclear) or ‘do not retrieve’. We retrieved the full texts of the study reports or publications, and two authors (S.‐H. Jeong and J.‐S. Min) independently screened the full text, identified studies for inclusion, and recorded the reasons for the exclusion of ineligible studies. Ultimately, 15 studies were selected for full‐text review.

**FIGURE 1:**
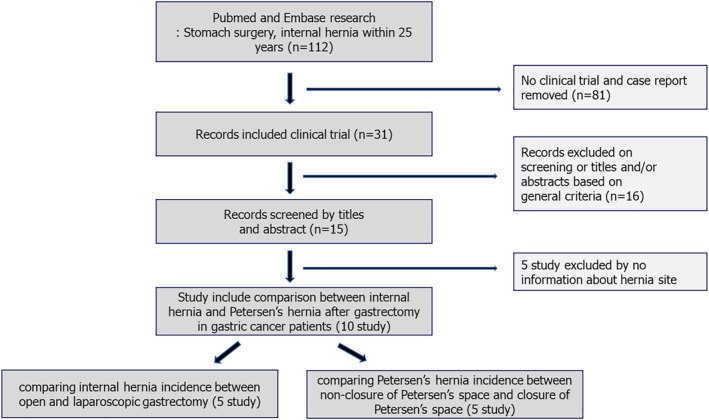
Flow diagram of the meta‐analysis.

Upon detailed evaluation, 5 studies were excluded because of the absence of specific information regarding the site of hernia formation. Consequently, 10 studies were included in the final qualitative and quantitative synthesis. These studies met the inclusion criteria as follows: (1) compared the incidence of IH following laparoscopic gastrectomy versus open gastrectomy or (2) evaluated the incidence of pH on the basis of whether Petersen's space was closed or left open during surgery. The included studies were further categorized into two subgroups. Five studies compared the incidence of internal hernia between laparoscopic and open gastrectomy approaches, whereas the other five studies investigated the incidence of Petersen's hernias between patients who did not undergo closure of Petersen's space and those who did undergo closure of Petersen's space. This selection process is illustrated in Figure 1 (see flow diagram).

### 2.2 Data Extraction

From the 5 selected studies, we extracted the following information: year of publication and study period, country and publication language of the study, sample size, baseline characteristics of the participants (age, sex, comparability between two groups, etc.), type of operation and reconstruction method, primary and secondary outcomes (number of internal hernias or Petersen's hernias), and follow‐up time after gastrectomy.

### 2.3 Assessment of Risk of Bias in Included Studies

Two authors (S.‐H. Jeong and J.‐S. Min) independently assessed the quality of the methodology of the included studies, following the quality checklist supplied in the Cochrane Handbook for Systematic Reviews of Interventions (Table 1); any discrepancies regarding the inclusion or exclusion of studies were resolved through discussion and final adjudication by a third senior reviewer (R.‐B Kim). This dataset contains six items: (1) random sequence generation, (2) allocation concealment, (3) blinding of participants, (4) incomplete outcome data, (5) selective outcome reporting, and (6) other biases. Considering the risk of overestimating intervention effects in randomized trials with inadequate methodological quality, we assessed the methodological quality of the trials according to the information available in the published articles. If this information was not available, we tried to contact the authors to request it. We reached consensus by discussion.

**TABLE 1:** Quality of methodology of the included studies, following the quality checklist supplied in the Cochrane Handbook for Systematic Reviews of Interventions.

1st author (Year)	Random sequence generation	Allocation concealment	Blinding of participants	Incomplete outcome data	Selective outcome reporting	Other bias
Yoshikawa K (2014) [5]	Low risk	Low risk	High risk	High risk	Unclear	Unclear
Ojima T (2017) [6]	Low risk	Low risk	High risk	High risk	Unclear	Unclear
Han WH (2019) [4]	Low risk	Low risk	High risk	High risk	Unclear	Unclear
Kang KM (2019) [2]	Low risk	Low risk	High risk	High risk	Unclear	Unclear
Ahn HM (2021) [7]	Low risk	Low risk	High risk	High risk	Unclear	Unclear

*Note:* Risk of bias domains were evaluated following the Cochrane Handbook for Systematic Reviews of Interventions. “Unclear” indicates insufficient information to permit a judgment of either low or high risk.

### 2.4 Statistical Analysis of Data

We performed the meta‐analyses via R version 4.4.1 (R Core Team, 2025, http://www.r‐project.org) with the “meta” package. To assess the risk of IH in laparoscopic gastrectomy compared with that in open surgery gastrectomy and to assess the risk of pH in nonclosure of Petersen's space groups compared with that in closure of Petersen's space groups, we calculated the pooled odds ratio (OR) with a 95% confidence interval (CI). The pooled ORs were calculated via the DerSimonian and Laird random‐effects model [8] with the Peto method. This method is typically used in cases of rare events to minimize bias in the results.

We presented heterogeneity in the forest plot via the *Q* test [9]. We considered a *p* value of less than 0.10 to indicate statistically significant heterogeneity. We also employed the *I* ^2^ statistic to gauge the extent of heterogeneity, with 50% or more signifying substantial to considerable heterogeneity. In instances of statistical or clinical heterogeneity, or both, we interpreted the results with caution and investigated potential sources of heterogeneity. To address the issue of methodological heterogeneity, we considered both the study design and the risk of bias. We explored clinical heterogeneity by comparing the characteristics of participants, interventions, controls, outcome measures, and study designs in the included studies.

Subgroup analysis was used to identify homogeneous subgroups in studies of total patients who underwent gastrectomy. We intended to assess publication bias with a funnel plot and Egger's test [10]. However, as the number of included studies was less than 10, we did not present any funnel plots, as recommended in Chapter 10 of the Cochrane Handbook for Systematic Reviews of Interventions [11]. To assess the bias of the synthesized results and recalculate the bias‐adjusted effect size, we performed sensitivity analyses via Rucker's limit method [12]. The limit method accounts for the fact that the effect size and standard errors of studies are not independent when there are small‐study effects. Therefore, it is assumed that publication bias particularly affects small studies and that small studies will have a larger effect size than large studies.

## 3 Results

### 3.1 Overview of Comparative Research on Internal Hernia Following Gastrectomy in Patients With Gastric Cancer

A total of five studies were included in the meta‐analysis comparing the incidence of IH between open gastrectomy (OG) and laparoscopic gastrectomy (LG) in patients with gastric cancer. The key characteristics of the included studies are summarized in Table 2.

**TABLE 2:** Key features of studies comparing internal hernia outcomes after gastrectomy in patients with gastric cancer.

Year	1st author	Institution	Closure of Petersen's space	Resection extent	Reconstruction	Internal hernia incidence in OG versus LG	*p* value	Mean timing of IH occurrence after surgery (months)	Follow up period after surgery (months)	Petersen's Hernia/Total internal hernia (number of occurrence)
2014	Yoshikawa K [5]	Single center	No	DG or TG	RNY	6/250 (2.4%) versus 6/178 (3.4%)	NA	20.6	51, mean	5/12
2017	Ojima T [6]	Single center	Yes or No	TG	RNY	4/407 (1.0%) versus 6/122 (4.9%)	0.005	27.1	52, median	2/10
2019	Han WH [4]	Single center	No	DG or TG	BII, RNY	7/3672 (0.19%) versus 17/2105 (0.81%)	< 0.001	20.9	NA	15/24
2019	Kang KM [2]	Single center	NA	DG or TG	BII, RNY, uRY	12/930 (1.3%) versus 92/2880 (3.2%)	NA	14.7	60.3, median	27/59
2021	Ahn HM [7]	Single center	NA	DG or TG or PG	BII, RNY, DT	3/935 (0.3%) versus 17/1335 (1.3%)	0.019	28	NA	18/20

Abbreviations: BII, Billroth II; DG, distal gastrectomy; DT, double tract reconstruction; LG, laparoscopic gastrectomy; NA, not applicable; OG, open gastrectomy; PG, proximal gastrectomy; RNY, Roux‐en‐Y; TG, total gastrectomy; uRY, Uncut Roux‐en‐Y.

All the studies were conducted at single‐center institutions and were published between 2014 and 2021 [2, 4, 5, 6, 7]. The extent of gastrectomy varied among studies, with all including distal gastrectomy (DG) and total gastrectomy (TG), while one study [7] also included proximal gastrectomy (PG). The most commonly employed reconstruction methods were Roux‐en‐Y (RNY), Billroth II (BII), and uncut Roux‐en‐Y (uRY), with double tract (DT) reconstruction also reported in one study [7].

The incidence of IH was consistently greater in the laparoscopic groups than in the open groups across all the studies. For example, Kang KM et al. reported an incidence of 1.3% (12/930) in OG groups versus 3.2% (92/2880) in LG groups, whereas Han WH et al. reported a lower but still significant difference of 0.19% (7/3672) in OGs compared with 0.81% (17/2105) in LG groups [2, 4]. The *p* values indicating the statistical significance of these differences ranged from < 0.001 to 0.019 in the three studies that reported them. The time to IH occurrence after gastrectomy ranged from 14.7 to 27.1 months, although not all studies provided these data.

The reported follow‐up durations ranged from 51 to 60.3 months. pH was identified as the predominant subtype of IH in most cases. In particular, Ahn HM, et al. reported that the proportion of IHs that were PHs was 90% (18/20), whereas Han WH, et al. reported a proportion of 62.5% (15/24) [4, 7]. Closure of Petersen's space was variably performed; Ojima T et al. included both closure and nonclosure cases, whereas others either did not report it or explicitly stated that the space was not closed [6].

### 3.2 Comparison of the Incidence of Internal Hernia Between Open and Laparoscopic Gastrectomy in Patients With Gastric Cancer: Meta‐Analysis

**FIGURE 2:**
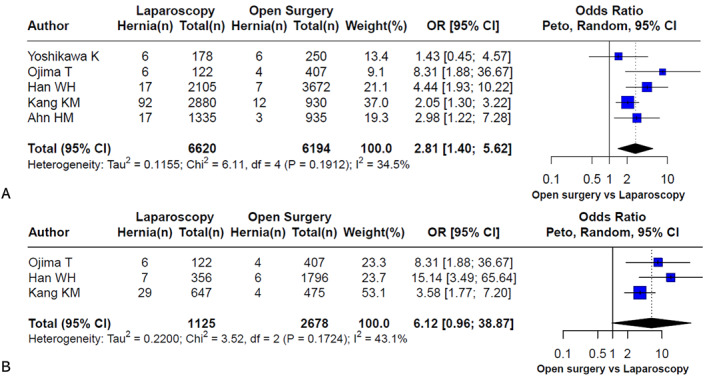
Meta‐analysis comparing the incidence of internal hernia after open gastrectomy versus that after laparoscopic gastrectomy in patients with gastric cancer. (A) Overall analysis including all types of gastrectomy (B) Subgroup analysis of patients who underwent total gastrectomy only.

A meta‐analysis was conducted to compare the incidence of IH following OG versus LG in patients who underwent gastrectomy for gastric cancer. The analysis results are summarized in Figure 2A,B.

Figure 2A presents pooled data from five studies including all types of gastrectomy—DG, TG, and PG. A total of 6620 patients underwent LG, and 6194 underwent OG. The incidence of IH was significantly greater in the laparoscopic group, with a pooled OR of 2.81 [95% CI: 1.40–5.62]. These findings suggest that LG is associated with a nearly threefold increased risk of IH compared with OG. Statistical heterogeneity was moderate (*I* ^2^ = 34.5%, *p* = 0.1912), indicating reasonable consistency across studies despite varying surgical types and reconstruction methods.

To specifically assess the risk in patients undergoing TG—a procedure inherently more susceptible to IH due to extensive mesenteric defects—a subgroup meta‐analysis was performed and is shown in Figure 2B. This analysis included three studies that reported the incidence of IH exclusively in patients who underwent TG (*n* = 1125 LG vs. 2678 OG). The pooled OR was markedly elevated at 6.12 [95% CI: 0.96–38.87], indicating a trend toward a greater risk in the laparoscopic group, although the result was not statistically significant (*p* ≈ 0.056). Heterogeneity was moderate (*I* ^2^ = 43.1%, *p* = 0.1724).

### 3.3 Effect of Petersen's Space Closure on Petersen's Hernia Incidence After Gastrectomy in Patients With Gastric Cancer: Comparative Summary

To evaluate the effect of Petersen's space closure on the incidence of pH following gastrectomy for gastric cancer, data from five studies published between 2014 and 2022 were analyzed (Table 3) [13, 14, 15, 16, 17]. These studies compared the incidence of pH between two groups: the nonclosure group (NCG) and the closure group (CLG) of Petersen's space. All the studies were conducted in Asian centers, with four being single‐center studies and one being a multicenter investigation involving 13 hospitals [16]. Most procedures were performed via laparoscopic approaches, and the majority of reconstructions utilized RNY anastomosis. The extent of gastrectomy varied, with both DGs and TGs included.

**TABLE 3:** Comparison of the incidence of Petersen's hernia according to nonclosure or closure of Petersen's space after gastrectomy in patients with gastric cancer.

Year	1st author	Institution	Approach	Resection extent	Reconstruction	Incidence of pH in NCG versus CLG	*p* value	Time intervals of pH after surgery	Follow up period after surgery (months)
2014	Kojima K [13, 14]	Single center	Laparoscopy only	DG	RNY only	6/268 (2.2%) versus 0/90 (0%)	0.06	11.5 months, mean	50.8
2017	Kimura H	Single center	Laparoscopy only	DG or TG	RNY only	7/250 (2.8%) versus 0/105 (0%)	0.06	NA	NA
2021	Pan T [15]	Single center	Laparoscopy or open	DG or TG	DG & BII, or TG & RNY	11/828 (1.3%) versus 1/385 (0.3%)	0.042	14.4 months, mean	NA
2022	Murakami K [16]	13 hospitals	Laparoscopy only	TG	RNY only	5/105 (4.8%) versus 3/609 (0.5%)	< 0.01	NA	NA
2017	Yamashita W [17]	Single center	Laparoscopy only	TG	RNY only	6/75 (8%) versus 0/47 (0%)	0.047	4.1 ± 0.8 years	52, median

Abbreviations: BII, Billroth II; CLG, closure group; DG, distal gastrectomy; NA, not applicable; NCG, non‐closure group; pH, Petersen's Hernia; RNY, Roux‐en‐Y; TG, total gastrectomy.

In every study, the nonclosure group presented a greater incidence of pH than did the closure group. Kojima et al. (2014) reported a difference in pH incidence: 6/268 in the nonclosure group versus 0/90 in the closure group (*p* = 0.06), with a mean time to pH diagnosis of 11.5 months after gastrectomy [14]. Kimura et al. (2017) reported 7 patients in the nonclosure group (7/250) and none in the closure group (0/105), which approached statistical significance (*p* = 0.06) [13]. Murakami et al. (2022), a large multicenter study, reported a significantly lower pH rate in the closure group (3/609 vs. 5/105, *p* < 0.01), strengthening the evidence for closure of Petersen's space [16]. Yamashita et al. (2017) also reported a significant benefit of closure (6/75 vs. 0/47, *p* = 0.047), with a notably long interval between gastrectomy and repeat surgery for pH (4.1 ± 0.8 years) [17]. Pan et al. (2021) reported that 1.0% (12/1213) of patients developed pH, and the rate of pH in the closure group was significantly lower than that in the nonclosure group (0.3%, 1/385 patients vs. 1.3%, 11/828 patients; *p* = 0.042) [15].

In most patients who developed pH across these studies, the mean follow‐up duration—when available—was approximately 50 months after gastrectomy. The average time to pH occurrence was generally approximately during the first year after surgery, with the earliest cases reported within 3 months, underscoring the importance of early anatomical closure to prevent acute IH events. Collectively, this evidence suggests that routine closure of Petersen's space during gastrectomy—particularly in laparoscopic total gastrectomy with RNY reconstruction—reduces the risk of Petersen's hernias.

### 3.4 Impact of Petersen's Space Closure on Petersen's Hernia Following Gastrectomy in Patients With Gastric Cancer: Meta‐Analysis

**FIGURE 3:**
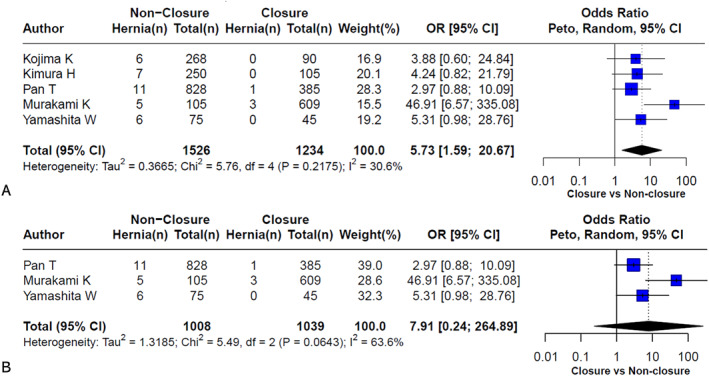
Meta‐analysis of Petersen's hernia incidence with and without Petersen's space closure in laparoscopic gastrectomy in patients with gastric cancer. (A) Overall analysis including all types of gastrectomy. (B) Subgroup analysis of patients who underwent total gastrectomy only.

To investigate whether closure of Petersen's space reduces the risk of pH following gastrectomy in patients with gastric cancer, a meta‐analysis was conducted, and the results are presented in Figure 3A,B.

Figure 3A summarizes the results of five studies including all types of gastrectomy, encompassing a total of 2760 patients (1526 patients in the nonclosure group vs. 1234 patients in the closure group), including patients who underwent DG and TG. The pooled analysis revealed a significantly greater risk of pH in the nonclosure group, with a combined OR of 5.73 [95% CI: 1.59–20.67]. This finding indicates that the odds of developing pH were nearly six times greater when Petersen's space was not closed. The heterogeneity among studies was low to moderate (*I* ^2^ = 30.6%, *p* = 0.2175), suggesting acceptable consistency across the included studies despite variation in surgical technique and reconstruction type.

The clinical benefit of Petersen's space closure was further quantified by calculating the Number Needed to Treat (NNT). Based on a representative incidence of Petersen's hernia (pH) of approximately 2.3% in the nonclosure group and 0.3% in the closure group, the absolute risk reduction (ARR) was 2.0%. This translates to an NNT of 50, indicating that approximately 50 patients need to undergo preemptive closure of Petersen's space to prevent one case of PH. This calculation highlights the strong clinical efficacy and justification for adopting routine closure as a standard preventive surgical step.

To specifically evaluate the risk of pH after TG, a subgroup analysis was conducted using data from three studies that clearly separated TG patients (Figure 3B). In this analysis, 2047 patients were included (1008 in the nonclosure group vs. 1039 in the closure group). The pooled OR was 7.91 [95% CI: 0.24–264.89], again favoring closure of Petersen's space, although the result did not reach statistical significance owing to the wide confidence interval and increased heterogeneity (*I* ^2^ = 63.6%, *p* = 0.0643). Notably, one study (Murakami et al.) reported a particularly large effect size, which substantially influenced the overall estimate and may have contributed to heterogeneity [16].

## 4 Discussion

This meta‐analysis was conducted to investigate whether laparoscopic gastrectomy is associated with a greater incidence of internal hernia than open gastrectomy is and to assess the effectiveness of prophylactic closure of Petersen's space in preventing Petersen's hernias. The findings demonstrated that laparoscopic approaches, particularly for total gastrectomy with Roux‐en‐Y reconstruction, significantly increase the risk of internal hernia, whereas routine closure of Petersen's space markedly reduces the incidence of Petersen's hernias and should be considered a standard preventive measure in minimally invasive gastric cancer surgery.

Compared with open surgery, laparoscopic gastrectomy is associated with a greater risk of internal hernia, primarily due to reduced postoperative adhesion formation [1, 13]. Adhesions in open surgery naturally limit bowel mobility, whereas laparoscopic techniques minimize postoperative intra‐abdominal adhesion, allowing intestinal loops to migrate into mesenteric defects [7]. Additionally, retrocolic reconstruction in laparoscopic procedures creates three potential herniation sites: Petersen's space, the jejunojejunostomy mesentery, and the transverse mesocolon, further increasing the degree of risk [3, 14, 18, 19, 20]. Patients undergoing laparoscopic total gastrectomy experience more significant postoperative weight loss compared to those undergoing laparoscopic distal gastrectomy. This excessive weight loss is thought to reduce mesenteric tension, consequently leading to a higher incidence of Petersen's hernia [3]. Laparoscopic total gastrectomy with Roux‐en‐Y reconstruction has a 4.5% (29/638) incidence of internal hernia following surgery, in contrast with 0.9% (9/1026) after open distal gastrectomy, highlighting the interplay between surgical extent and approach [2].

In bariatric surgery, internal hernia is a well‐recognized late complication after laparoscopic Roux‐en‐Y gastric bypass, and the magnitude of risk reported in that literature provides a useful benchmark for interpreting our findings in gastric cancer surgery. In a large multicenter randomized trial, the routine closure of mesenteric defects at both the jejunojejunostomy and Petersen's space reduced reoperation for small‐bowel obstruction, although it modestly increased early severe complications, primarily due to kinking at the jejunojejunostomy [21]. Long‐term follow‐up of the same randomized cohort demonstrated sustained benefit with a lower incidence of reoperation for small‐bowel obstruction and a marked reduction in reoperation for internal herniation [22]. Similarly, a single‐center randomized trial using clip closure reported a lower internal herniation rate with closure [23]. Consistent with these trials, a systematic review and meta‐analysis in laparoscopic Roux‐en‐Y gastric bypass reported lower internal hernia rates when mesenteric defects were closed [24]. These bariatric data align with the mechanistic rationale in gastrectomy (reduced adhesions and postoperative loss of mesenteric fat increasing bowel mobility) and support routine, meticulous defect closure as a preventive strategy, while also emphasizing the importance of technical standardization to minimize kinking‐related early obstruction.

Prophylactic closure of mesenteric defects via nonabsorbable sutures reduces the incidence of internal hernia [2, 6, 13]. Retrocolic reconstruction mandates closure of Petersen's space, jejunojejunostomy mesentery, and transverse mesocolon defects, whereas antecolic methods require only Petersen's space closure [2, 16, 20]. Standardized techniques, such as continuous barbed sutures, improve the reliability of defect closure [3]. Routine closure of Petersen's space decreases hernia incidence even in laparoscopic total gastrectomy [2]. Early intervention within 24 h of symptom onset reduces bowel necrosis rates, emphasizing the importance of rapid diagnosis via CT whirlpool sign detection [17]. Postoperative weight management is critical, as excessive weight loss correlates with an increased risk for internal hernia after gastrectomy due to mesenteric fat loss [2, 25, 26, 27, 28].

Although closure of Petersen's space with nonabsorbable sutures is the standard preventive approach, alternative strategies have been explored because of concerns about incomplete closure, recurrence, or technical difficulties, including those associated with mesenteric bleeding. These adjunctive methods focus on anatomical fixation or reinforcement to eliminate the risk of internal herniation through Petersen's space. First, jejunal mesentery fixation (Mefix method) involves suturing the jejunal mesentery (approximately 30 cm below the jejunojejunostomy) to the transverse mesocolon via nonabsorbable barbed sutures [29, 30]. Compared with traditional closure of Petersen's space, this method not only reduces the operative time—averaging just under four minutes—but also appears to eliminate the occurrence of Petersen's hernias in short‐term follow‐up studies. Second, the BIO synthetic mesh reinforcement approach reinforces a sutured Petersen's defect with a small bioabsorbable synthetic mesh, which is secured in place using surgical glue [31]. This method dramatically reduced internal hernia rates, which decreased from approximately 14% with glue‐only closure to less than 1% with mesh reinforcement. The third alternative method is jejunal loop fixation [32, 33]. In this method, the biliopancreatic limb is anchored to the transverse mesocolon, preventing the bowel from migrating into Petersen's space even if the space itself is left open. This technique has demonstrated success even in superobese patients, and it does not create additional mesenteric defects during fixation. Traditional closure of Petersen's space is highly effective, but alternative methods such as the Mefix technique, mesh reinforcement, and jejunal loop fixation offer comparable or even superior prevention rates. The Mefix method in particular is notable for its speed and safety, whereas mesh reinforcement provides added security for patients at high risk of hernia. Jejunal loop fixation is especially useful in complex or high‐risk cases. A combination of these techniques, such as the use of nonabsorbable sutures with adjunctive mesh or fixation, may provide the most robust prevention against Petersen's hernias.

While various techniques such as continuous suturing or clip application can be used to close Petersen's space, understanding the risk factors is equally crucial. Factors such as significant postoperative weight loss, which reduces mesenteric fat, may increase the potential space for herniation, necessitating a more proactive approach [2]. Additionally, it would be impractical to mandate regular CT follow‐ups for all patients. However, we believe that immediate radiological evaluation is essential for patients who experience rapid weight loss after laparoscopic gastrectomy if clinical symptoms such as abdominal pain or vomiting occur. This meta‐analysis had several limitations. First, most included studies were retrospective and single‐center studies, which may have introduced selection bias and limit the external validity (generalizability) of the results, as findings from such studies may not be representative of broader or more diverse patient populations. The retrospective design also increased the risk of selection bias; the process of selecting exposed and nonexposed groups may be related to the outcome, and differences in data completeness or record retention can further skew the results. Second, data incompleteness or missing outcome data in the original studies can introduce further bias and uncertainty, potentially affecting the accuracy of the pooled estimates in the meta‐analysis. Heterogeneity was notable in the subgroup analyses, especially for total gastrectomy. Moreover, the variability in reporting Petersen's space closure and follow‐up duration limits the consistency and generalizability of the findings. Third, we acknowledge the clinical concern that incomplete closure of Petersen's space may paradoxically lead to complications, such as a higher risk of bowel resection due to strangulated ileus. Fourth, the inclusion of predominantly Asian studies is attributed to the high regional incidence of gastric cancer and the standardized use of Roux‐en‐Y reconstruction. Fifth, data on robotic gastrectomy were unavailable for this meta‐analysis due to a lack of comparative studies focusing on internal hernia outcomes in this specific modality. However, a formal meta‐analysis on the rate of strangulation or bowel resection specifically resulting from technically inadequate or incomplete closure of the defect could not be conducted. This is due to the inherent data insufficiency and lack of granularity in the original studies, as most do not report detailed information on the quality of defect closure or the specific etiology of the hernia. However, a major strength of this meta‐analysis was its comprehensive evaluation of internal hernia incidence and Petersen's hernia incidence after gastrectomy in patients with gastric cancer, including subgroup analyses based on surgical approach, extent of resection, and Petersen's space closure, which enhances its clinical relevance and applicability to current surgical practice.

In conclusion, laparoscopic gastrectomy is associated with an increased risk of internal hernia compared to open surgery for gastric cancer. The preventive closure of Petersen's space should be considered mandatory, particularly when Roux‐en‐Y reconstruction is performed after gastrectomy.

## Author Contributions

**Sang‐Ho Jeong:** conceptualization, methodology, validation, formal analysis, investigation, resources, data curation, writing – original draft, writing – review and editing, visualization, supervision. **Rock Bum Kim:** methodology, software, validation, formal analysis, investigation, resources, data curation, writing – original draft, writing – review and editing, visualization. **Miyeong Park:** conceptualization, writing – review and editing. **Kyung Won Seo:** conceptualization, writing – review and editing. **Jae‐Seok Min:** conceptualization, methodology, validation, formal analysis, investigation, resources, data curation, writing – original draft, writing – review and editing, visualization, supervision.

## Funding

The authors have nothing to report.

## Conflicts of Interest

The authors declare no conflicts of interest.

## Data Availability

The data that support the findings of this study are available on request from the corresponding author. The data are not publicly available due to privacy or ethical restrictions.
